# Sublingual Sufentanil vs. Intravenous Fentanyl for the Treatment of Acute Postoperative Pain in the Ambulatory Surgery Center: A Randomized Clinical Trial

**DOI:** 10.1155/2022/5237877

**Published:** 2022-07-08

**Authors:** Aaron Berg, Jason Habeck, Michael Strigenz, Jonah Pearson, Alexander Kaizer, Jacob Hutchins

**Affiliations:** ^1^Department of Anesthesiology, University of Minnesota Medical School, Minneapolis, MN, USA; ^2^Department of Biostatistics and Informatics, Colorado School of Public Health, University of Colorado, Aurora, CO, USA

## Abstract

**Objectives:**

Sublingual sufentanil is a novel opioid medication to treat moderate to severe pain postoperatively. This study's aim was to determine if a single dose of a sublingual sufentanil tablet (SST) is as efficacious as a single dose of intravenous (IV) fentanyl in readiness to discharge from ambulatory surgery.

**Methods:**

This was a two-arm, parallel group, randomized prospective outcomes study conducted at a single, free-standing ambulatory surgery center. Patients aged 18–80 undergoing general anesthesia who developed a postoperative pain score of ≥ 4 were enrolled and randomized to receive either 30 mcg SST or 50 mcg IV fentanyl. After their initial randomized dose, rescue IV fentanyl followed by oral oxycodone if needed. Recovery length of stay from arrival in the postanesthesia care unit until readiness to discharge criteria was met based on phase 2 discharge criteria.

**Results:**

75 patients were analyzed. Readiness to discharge from the recovery room was not significantly different between either group (IV fentanyl median 65 minutes; IQR 56–89; SST 73 min, IQR 58–89; *p*=0.903). There was no significant difference in the amount of morphine equivalents (MME) of rescue opioids needed (IV fentanyl median rescue MME of 22.5, IQR 13.1–23.4; SST median rescue MME of 15.0, IQR 7.5–30.0; *p*=0.742). The change in pain from PACU initially, and on discharge was not significantly different (IV fentanyl initial pain minus pain on discharge median 3, IQR 2–4; SST initial pain minus pain on discharge median 4, IQR 2–5.5; *p*=0.079). There was no difference in the six-item screener and the Overall Benefit of Analgesic Survey Score. *Discussion*. In conclusion, patients who received a sublingual sufentanil 30 mcg tablet had no significant differences in PACU length of stay or rescue analgesic usage when compared to intravenous fentanyl 50 mcg.

## 1. Introduction

There is an increasing shift of surgical procedures towards the ambulatory surgery landscape resulting in the development of multi-specialty ambulatory surgery centers (ASCs). Postoperative pain continues to be one of the main factors that can prolong a patient's time in the postanesthesia care unit (PACU) [1]. Up to 41% of patients in the PACU report moderate to severe pain [2]. Effectively treating a patient's pain, while minimizing adverse events, is the goal of any effective analgesic regimen in the PACU.

Intravenous (IV) fentanyl is a commonly administered analgesic medication in the PACU given its rapid onset and no active metabolites. However, fentanyl has a short duration of action and often requires redosing at frequent intervals [3]. Sublingual sufentanil (SST 30 mcg; DSUVIA, AcelRx, Redwood City, CA, USA) is a lipophilic opioid that has been shown to be effective in treating moderate to severe pain postoperatively and in emergency departments [3]. It is available in a single dose 30 mcg tablet. SST has no active metabolites and does not require dose adjustments [4]. Previous studies have illustrated an onset of action of 15 minutes and analgesia lasting approximately 2 to 3 hours before requiring redosing [3, 4]. A pooled phase III safety analysis has discovered that SST 30 mcg is equivalent to intravenous morphine 5 mg [5]. The most common side effects include nausea, headache, and dizziness [3–5].

While SST has shown to be effective compared to placebo it has yet to be compared to intravenous fentanyl. Our hypothesis was that the use of SST would lead to improved pacu times. The primary objective of this study was to determine if a single dose of SST would lead to a shorter PACU length of stay when compared to IV fentanyl for patients undergoing a general anesthetic in an Ambulatory Surgery Center. Secondary outcomes were to evaluate the effectiveness in treating acute postoperative pain and the incidence of side effects.

## 2. Methods

This was a two-arm, parallel group, randomized prospective outcomes trial conducted at a single, free-standing ASC. The study was approved by the University of Minnesota institutional review board on December 2^nd^, 2019, and written informed consent was obtained from all participants (Principal Investigator: Aaron Berg; IRB STUDY00007956; clinicaltrials.gov identifier NCT04177862, date of registration 11/22/2019, date of first patient enrollment 12/11/2019, https://clinicaltrials.gov/ct2/show/NCT04177862). This manuscript adheres to the applicable CONSORT guidelines.

### 2.1. Primary Inclusion and Exclusion Criteria

All patients undergoing general anesthesia were screened and approached for study participation upon arrival at the ambulatory surgery center. English-speaking, male, and nonpregnant female patients aged 18–80 years were eligible for inclusion. Patients were excluded for opioid tolerance (defined as daily opioids for 3 or more months), known allergy to fentanyl or intolerances to the study medications, or undergoing cataract and oncologic procedures.

### 2.2. Study Treatment and Rescue Medication

Consented patients were given a multimodal analgesic regimen consisting of 975 mg acetaminophen and 300 mg of gabapentin if under the age of 65 (patients older than 65 only received acetaminophen) pre-operatively. Intraoperative pain medication was not protocol driven, but limited to only IV fentanyl and/or ketorolac. Once in PACU, patients with a postoperative pain intensity score of ≥ 4 on an 11-point numeric rating scale (NRS; 0 = no pain; 10 = worst imaginable pain) during PACU phase 1 recovery were randomized to receive either 30 mcg SST or 50 mcg IV fentanyl. Rescue medication, if necessary, was given to both groups according to the following protocol. After a minimum of 10 minutes from the initial study treatment drug, if pain intensity remained ≥ 4, 25 mcg pushes of IV fentanyl up to every 3 minutes for a max dose of 100 mcg was given. If pain remained ≥ 4 after this, 5 mg of oral oxycodone (or 2 mg of oral hydromorphone if allergic to oxycodone) was given. Once phase 1 criterion was met as defined by achieving an Aldrete score of 8 or higher, the patient was moved to phase II. Then in phase 2, the patient was only given an oral opioid if their indicated pain intensity score was ≥ 4. No IV fentanyl was given in phase 2.

### 2.3. Outcomes and Assessments Included in Analysis

The primary endpoint was the time to readiness for discharge after arrival in PACU, as defined in minutes by the period “In phase 1” to “Phase 2 recovery discharge criteria met.” This was defined as a phase 2 modified Aldrete score of greater or equal to 18, as well as a temperature of at least 36 degrees Celsius as per our ambulatory center's guidelines. The secondary endpoint was the amount of rescue opioids given until discharge. These medications were compared independently and also converted to milligram morphine equivalents (MME) using https://www.globalrph.com for overall opioid comparison. [6].

For cognitive assessment, a six-item screener was performed twice, first preoperatively during the study consent process and again near the one-hour point after study medication intervention (SST or IV fentanyl administration). [7] This six-item screener consists of asking the patient to remember three words, then asking three orientation questions followed by assessing recall of the original three words. Once a discharge criterion was met, a 7-item survey that assesses pain intensity and opioid-related adverse effects, the Overall Benefit of Analgesic Score (OBAS), was administered by the PACU nurse or research assistant. [8] Additional secondary endpoints included monitoring for opioid-related adverse events (AEs), such as postoperative nausea and vomiting (PONV), and any supplemental oxygen therapy to keep oxygen saturation above 93% used past 30 minutes into the PACU stay.

### 2.4. Power Analysis

Using internal, nonpublished, 2019 third quarter (Q3) ASC discharge data, we determined the sample sizes that would be needed to achieve different levels of power for a randomized study assigning participants equally to either the standard of care or a new intervention. In total, 598 cases in our Q3 data were similar to our targeted cases, with a mean PACU duration of 85 minutes (standard deviation of 33 minutes). Assuming a two-sided two-sample *t*-test with *α* = 0.05, the expected sample size needed to achieve an absolute reduction of 30 minutes in time for a 95% statistical power was calculated to be 33 per group.

### 2.5. Randomization

Permuted block randomization with sizes 2, 4, 6, and 8 possible (with probabilities of 35%, 35%, 20%, and 10%, respectively) was used to develop the random allocation sequence. If a participant that had consented to the study proceeded to meet enrollment pain intensity criteria, as assessed by the PACU nurse, that nurse would be informed of the randomization arm by a research assistant, and the assigned medication was administered.

### 2.6. Statistical Methods

Demographic summary measures are presented as median (interquartile range) and as count (percent) for continuous and categorical measures, respectively. Continuous outcomes were assessed for normality using the Shapiro–Wilk test. Since most continuous outcomes were not normally distributed, the Mann-Whitney nonparametric *U* test was used to compare randomized groups, with the median (interquartile range) presented for summary statistics. Fisher's exact test was used to compare categorical variables between the randomized groups. All tests are two-sided unless otherwise stated. *p*-values <0.05 are considered significant and are not adjusted for multiple comparisons. Data were analyzed as intention-to-treat. All analyses were completed with R version 3.6.3 (Vienna, Austria).

## 3. Results

### 3.1. Patient Disposition, Demographics, and Covariates

**Figure 1:**
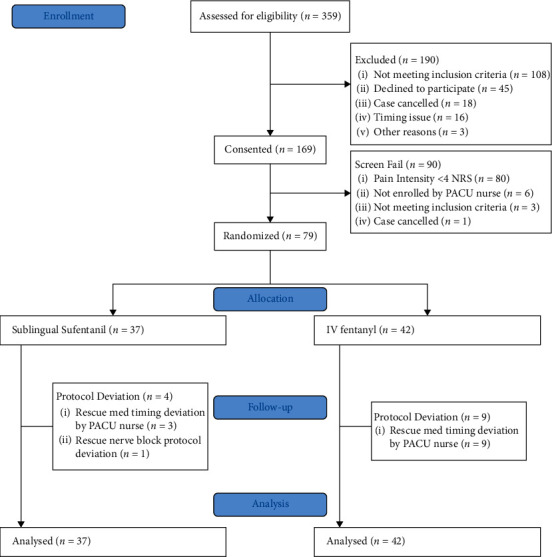
CONSORT diagram.

We consented to 169 patients from December 11^th^, 2019 through January 31^st^, 2020. Of this, 80 patients that did not achieve a pain score of 4 or greater in the PACU, 9 were given pain medications prior to potential randomization, 3 procedures were changed from general to a MAC, 1 was recognized as a screening failure after being consented and 1 case was canceled postinduction of anesthesia and hence were not eligible for randomization. In total, 75 patients were randomized with 9 not completing the study due to nurse protocol violations, yet those patients were still included in the final analysis with an intention to treat. 35 were randomized to sublingual sufentanil and 40 randomized to the fentanyl group with 33 in each group fully completing the study protocol (Figure 1).

Baseline demographics were not significantly different between the two groups, save for surgical duration, which was longer, respectively, in the IV fentanyl group compared to the SST group and age, which was younger, respectively, in the IV fentanyl group (Table 1). The most common surgical subspecialty procedure for each group was orthopedic surgery (42.5% in the fentanyl group and 54.3% in the SST group) but a wide range of surgical subspecialties was enrolled (Table 2).

### 3.2. Study Outcomes

The length of time until readiness for discharge from the recovery room was not significantly different between SST (73 minutes IQR 56–89) vs. IV fentanyl (65 minutes IQR 56–89) (*p*=0.903) (Table 3). 100% of patients in the fentanyl group and 97% of patients in the SST group (one refused) received either oral acetaminophen or a combination of oral acetaminophen and oral gabapentin pre-operatively. Thirteen patients (32.5%) in the fentanyl group and six patients (17.1%) in the SST group (*p*=0.184) received a preoperative nerve block. Intraoperatively there was no significant difference in the amount of fentanyl or ketorolac given between the two groups.

There was a trend towards but no significant difference when assessing the change in pain from initially presenting to the PACU and pain on discharge (Table 3). However, there was no significant difference between the two groups in the amount of rescue opioids in MME needed after their initial dose of SST or IV fentanyl. 7 patients in the SST group vs. 6 patients in the IV Fentanyl group did not receive rescue medications (*p*=0.791). The average rescue dose given to the SST group was 2.42 which was similar to the IV fentanyl group of 2.66 doses; both groups had a median of 2 doses. There was also no difference in OBAS scores between the two groups (Table 3).

There were no major postoperative adverse events recorded. Seven patients in the IV Fentanyl group (17.5%) and four in the SST group (11.4%) had postoperative nausea and vomiting (*p*=0.528). Four patients (11.4%) in the SST group and four patients (10.0%) in the IV fentanyl group required oxygen for greater than 30 minutes in the recovery room (*p*=1.0). There were no major respiratory desaturation events (oxygen saturation <90%) recorded. There was no difference between the two groups in cognition as assessed via a six-item screener at baseline, but a lower proportion of SST at 1-hour poststudy drug administration reported scores less than six (82.9% vs. 97.4%, *p*=0.047) (Table 4).

## 4. Discussion

This is a prospective active comparator trial involving the 30 mcg sublingual sufentanil tablet. This trial was designed with a high power to detect if SST produced a meaningful reduction in PACU duration when substituted for IV fentanyl for the initial treatment of moderate-to-severe acute postoperative pain. The goal of this study was for a generalized real-world application of a novel medication to see if its initial use would have a benefit over standard of care, in this case, IV fentanyl. This study showed no statistically significant difference in PACU times. Both groups' PACU duration fell within our normal range for general anesthetics and those times were consistent with other reported PACU times at multi-specialty ambulatory surgery centers [9]. The average length of stay in PACU at our ASC is on the lower end of the average range in those previous studies. The similar PACU times may be related to the fact that the duration of time spent in PACU is multifactorial and things unrelated to pain or opioid side effects kept patients from being ready to discharge. Things like postoperative nausea and vomiting, age, residual anesthesia, or the procedure performed could have led to a longer PACU stay, and as such it may be that there is little room for improvement in PACU time with any opioid intervention in the PACU. Furthermore, the IV fentanyl group had longer surgical procedure times which also may have impacted the duration of PACU time between the two groups.

Our study also demonstrated no significant difference in the need for additional rescue medication (either in the amount of rescue medications needed or the number of patients who did not need rescue medications) or pain scores on discharge after receiving either SST or IV fentanyl. It is important to note that our study terminated upon readiness to discharge from the PACU, and thus only looked at roughly the first 60–70 minutes after dosing. With the *T* max of the 30 mcg dose of SST being 1 hour, it is possible that by terminating the study at readiness to discharge the full analgesic comparison between the two medications was not completely assessed. [10] The SST group trended toward a larger decrease in pain from when first presenting to the PACU and thus may illustrate further need to study the analgesic benefit beyond discharge from the PACU. Additionally, SST was only substituted for the initial dose and therefore any possible benefit for redosing was not assessed during this trial.

Adverse events were similar between the two groups. The rate of nausea and vomiting (11.4%) was lower than previously described for SST as the study by Minkowitz et al. showed a nausea rate of 29.0% in the SST group [11]. However, all patients at our ASC undergoing general anesthesia receive 2 or 3 intraoperative prophylactic antiemetics and the use of multimodal analgesia to decrease overall opioid requirements and PONV. The two groups had a similar incidence of patients requiring oxygen for greater than 30 minutes and no patients in either group had recorded SpO_2_ values below 90%. Thus, both interventions demonstrated minimal respiratory adverse effects at the doses given. Furthermore, when assessing cognition via the six-item screener (SIS) tool between the two groups there again was no significant difference thus suggesting neither intervention had a negative effect on patient cognition one hour after an intervention. This echoes the previous study by Miner et al. evaluating cognition after dosing SST in the emergency department yielded similar results [12].

While no other studies were found comparing SST 30 mcg to an active comparator, there was one previous study by Melson et al., which compared a different formulation of sublingual sufentanil (SST 15 mcg; Zalviso®, Grünenthal GmbH, Aachen, Germany) to an active comparator. In that study, sublingual sufentanil was supplied via a handheld PCA at a dose of 15 mcg with a 20 minutes lockout [13]. That formulation of SST was compared to IV morphine 1 mg with a lockout of 6 minutes. SST was shown to have noninferiority at the 48-hour time point of Patient Global Assessment of the method of pain control and SST provided more rapid analgesia and higher patient and nurse satisfaction. Using the data from Miner et al. determined the morphine equivalent of SST [5]. In the first five hours after treatment was initiated, it was found that SST 15 mcg was equal to 2.5 mg of IV morphine. This equivalency is further illustrated in our study as we found the SST 30 mcg to be similar to 50 mcg of IV fentanyl.

We do note several limitations in our study. The first limitation was that because of the two different routes of administration, the nurse giving the medications and the patient emerging from anesthesia were nonblinded to the intervention. Additionally, while not significantly different, the multimodal pain protocol was not standardized intra-operatively. Finally, the patients were not all with the same PACU nurse. While a protocol for when rescue opioids were to be given and withheld, this variable could have added a bias to the study.

Future studies could evaluate the analgesic benefit of SST beyond discharge from PACU in an ambulatory setting. Since our study was terminated upon meeting phase 2 discharge criteria, a complete comparison could have been missed. In addition, this study focused on the initial use and not comparing only SST to IV Fentanyl. Alternatively, the timing of SST either preoperatively or intraoperatively could be studied to see if earlier administration could decrease PACU time, reduce pain scores, or opioid use in PACU.

## 5. Conclusion

In conclusion, patients who received a sublingual sufentanil 30 mcg tablet had no significant differences in PACU length of stay or rescue analgesic usage when compared to intravenous fentanyl 50 mcg.

## Figures and Tables

**Table 1:** Demographics.

	Fentanyl (median (IQR) or *N* (%)) *N* = 40	Sublingual sufentanil median (IQR) or N (%) *N* = 35
Male	16 (40.0%)	13 (37.1%)
Age (years)	37.0 (22.8, 53.8)	52.0 (32.5, 62.0)
Weight (kilograms)	77.4 (68.9, 97.6)	79.4 (69.0, 98.7)
BMI (kg/m^2^)	29.9 (24.3, 33.6)	28.3 (24.3, 31.6)
Surgical duration (minutes)	76.5 (33.0, 113.3)	45.0 (31.0, 68.5)
ASA I	14 (35.0%)	12 (34.3%)
ASA II	21 (52.5%)	15 (42.9%)
ASA III	5 (12.5%)	8 (22.9%)

IQR: interquartile range, kg: kilogram, m: meters, *N*: number of patients. Mann–Whitney nonparametric *U* test and Fisher's exact test was used to compare groups for continuous and categorical measures, respectively.

**Table 2:** Surgical subspecialties enrolled in the study.

Surgical subspecialty	Fentanyl N (%) *N* = 40	Sublingual sufentanil N (%) *N* = 35
General surgery	2 (5.0%)	1 (2.9%)
Minimally invasive	1 (2.5%)	2 (5.7%)
Oculoplastic	1 (2.5%)	1 (2.9%)
Ophthalmology	1 (2.5%)	0 (0%)
Orthopedics	17 (42.5%)	19 (54.3%)
Otolaryngology	7 (17.5%)	6 (17.1%)
Plastics	6 (15.0%)	3 (8.6%)
Surgical oncology	1 (2.5%)	1 (2.9%)
Urology	4 (10.0%)	2 (5.7%)

*N*: number, (a). *p*-value compares sublingual sufentanil vs. IV fentanyl, (b). Mann–Whitney nonparametric *U* test was used to compare medians and chi-squared test was used to compare proportions.

**Table 3:** Recovery room data.

	Fentanyl median (IQR) *N* = 40	Sublingual sufentanil median (IQR) *N* = 35	*p*-value
Phase 1 to phase 2 discharge criteria met (min)	65 (56, 89)	73 (56, 89)	0.903
Initial pain score in PACU	6.0 (5.0, 7.0)	7.0 (5.5, 8.0)	0.117
Max pain in PACU	7.0 (5.0, 8.0)	7.0 (6.0, 8.8)	0.313
Pain at discharge	3.0 (2.0, 4.0)	3.0 (2.0, 4.0)	0.198
Initial pain minus pain at discharge	3.0 (2.0, 4.0)	4.0 (2.0, 5.5)	0.079
Rescue fentanyl (mcg)	50.0 (18.8, 50.0)	50.0 (25.0, 87.5)	0.470
Rescue oxycodone (mg)	5.0 (0.0, 5.0)	0.0 (0.0, 5.0)	0.028
Rescue MME	22.5 (13.1, 23.4)	15.0 (7.5, 30.0)	0.742
OBAS score	3.0 (1.0, 4.5)	3.0 (2.0, 4.0)	0.826

IQR: interquartile range, mcg: micrograms, mg: milligrams, MME: milligram morphine equivalent, min: minutes, *N* number, *p*-value compares sublingual sufentanil vs. IV fentanyl,

Mann–Whitney nonparametric *U* test was used to compare groups.

**Table 4:** Six item screener scores.

Outcome	Score	*N* (%) fentanyl	*N* (%) SST	*p*-value
Preop six item screener score	3	1 (2.5%)	0 (0.0%)	1.000
	4	0 (0.0%)	0 (0.0%)	
	5	2 (5.0%)	2 (5.7%)	
	6	37 (92.5%)	33 (94.3%)	
1-hour post medication six item screener score	4	0 (0.0%)	2 (5.7%)	0.081
	5	1 (2.6%)	4 (11.4%)	
	6	38 (97.4%)	29 (82.9%)	

*N*: number, *p*-value compares sublingual sufentanil vs. IV fentanyl, Chi-squared test was used to compare groups.

## Data Availability

The data sets generated and analyzed during the current study are not publicly available due to the IRB but are available from the corresponding author on reasonable request.
